# HDPose: Post-Hierarchical Diffusion with Conditioning for 3D Human Pose Estimation

**DOI:** 10.3390/s24030829

**Published:** 2024-01-26

**Authors:** Donghoon Lee, Jaeho Kim

**Affiliations:** 1 Department of Information and Communications Engineering, Sejong University, Seoul 05006, Republic of Korea; jupiter8790@sju.ac.kr; 2 Department of Electrical Engineering, Sejong University, Seoul 05006, Republic of Korea

**Keywords:** 3D human pose estimation, diffusion, transformer, hierarchical structure

## Abstract

Recently, monocular 3D human pose estimation (HPE) methods were used to accurately predict 3D pose by solving the ill-pose problem caused by 3D-2D projection. However, monocular 3D HPE still remains challenging owing to the inherent depth ambiguity and occlusions. To address this issue, previous studies have proposed diffusion model-based approaches (DDPM) that learn to reconstruct a correct 3D pose from a noisy initial 3D pose. In addition, these approaches use 2D keypoints or context encoders that encode spatial and temporal information to inform the model. However, they often fall short of achieving peak performance, or require an extended period to converge to the target pose. In this paper, we proposed HDPose, which can converge rapidly and predict 3D poses accurately. Our approach aggregated spatial and temporal information from the condition into a denoising model in a hierarchical structure. We observed that the post-hierarchical structure achieved the best performance among various condition structures. Further, we evaluated our model on the widely used Human3.6M and MPI-INF-3DHP datasets. The proposed model demonstrated competitive performance with state-of-the-art models, achieving high accuracy with faster convergence while being considerably more lightweight.

## 1. Introduction

The goal of monocular 3D human pose estimation (HPE) is to localize 3D body joints from 2D images or video. This task is crucial in computer vision applications, such as human–robot interaction [1], autonomous driving [2], the metaverse [3], and VR [4]. In recent years, coupled with the success of deep learning, the performance of monocular 3D HPE has made notable progress [5,6,7,8,9]. The lifting-based approach [10] involves mapping from a 2D pose to a 3D pose. This approach does not estimate the 3D pose directly from the image, thereby achieving high performance with less influence from background and lighting conditions. The dilated temporal-based approach [11] uses a fully convolutional model dependent on dilated temporal convolutions over 2D keypoints to effectively estimate 3D poses in videos. By learning the trajectory of a 2D keypoint’s movement over time, this approach facilitates the forecasting of the sequential movement of each joint within the human anatomy across successive frames of a video. Furthermore, the transformer-based approach [7] was proposed to learn the temporal trajectories by capturing the surrounding sequences and the long-range associations of the input sequence. These approaches have made significant contributions to improving the performance of 3D HPE. By leveraging the capabilities of these methods, the accuracy and reliability of pose estimation have been considerably improved. However, despite advancements in 3D HPE, monocular 3D HPE faces challenges in terms of depth ambiguity and various occlusion scenarios, which result in the reconstruction of incorrect 3D poses.

To overcome these limitations, previous studies have proposed methods utilizing diffusion models. Diffusion models have facilitated the realization of imposing performance. Representatively, denoising diffusion probabilistic models (DDPMs) [12] have rendered image generation plausible by gradually denoising sampled data from a Gaussian distribution. Intuitively, the DDPM mechanism entails treating the incorrect data in 3D poses, which are caused by occlusion, as noise. Based on the gradual denoising of this noise via small iterative steps, the correct 3D pose can ultimately be converged. However, reconstructing the correct 3D pose using only the incorrect 3D pose sampled from the pure a Gaussian distribution is challenging owing to the lack of sufficient evidence to predict the correct 3D pose from an incorrect one. Moreover, the learning process for reconstructing the correct 3D pose is time-consuming owing to the numerous steps involved in the denoising process. Therefore, in the denoising process, the conditions are vital for providing guidance for the transition from an incorrect 3D pose towards the correct 3D pose. This study focused on manipulating conditions to guide 3D poses more accurately and quickly.

Currently, 3D HPE using diffusion involves two distinct methods for providing conditions. The first approach [13] is the provision of a simple 2D pose. This method provides evidence through a 2D pose, thus enabling the 3D pose sampled from pure Gaussian noise to be guided towards the correct 3D pose. However, a simple 2D pose does not yield high performance, as it cannot capture the temporal information between frames in a 2D pose sequence. The second approach [14] is a context encoder that includes spatial and temporal information and can outperform simple 2D poses. This method can outperform the first approach because it captures correlations in 2D pose joints and frames. However, this method requires more training steps to converge to optimal performance, resulting in longer training times.

To address the issues highlighted, this study proposed a hierarchical diffusion 3D human pose estimation (HDPose). We aimed to learn to guide the denoising model to clean the 3D pose in a precise and rapid manner by passing detailed spatial and temporal information to it in a hierarchical structure. We were inspired by the method proposed by Lu et al. [15], wherein the HDAE [15] exploited the low-level-to-high-level feature hierarchy. This method can efficiently and deterministically transform a semantic image from Gaussian noise. Therefore, we designed a hierarchical conditioning model inspired by the above literature. The proposed method utilized the context encoder described previously. This effectively encapsulated the context encoder of the final layer output, which integrated skip connections and spatio-temporal features, thereby seamlessly capturing both spatial and temporal features at diverse scales. Consequently, we hierarchically aggregated the last features extracted from the conditional model to each encoder of the denoising model. This enabled the neural network to learn more efficiently and converge rapidly. We compared the performance of various methods that feed features from the condition model to the denoising model. Empirically, we found that the most effective approach was to hierarchically aggregate the holistic representation produced by the final encoder of the condition. In addition, compared to previous models [13,14], we observed that we achieve significantly better performance by using a pre-trained condition model.

We experimented with different conditional model structures, including simple 2D pose sequences, non-hierarchical structure, pre-hierarchical structure, and post-hierarchical structure. Our model was easily trainable and could more accurately predict 3D poses that matched 2D poses with high-quality poses. Moreover, it quickly converged to the optimal 3D pose during the learning process and was a more lightweight model than the previous approach [7,16]. In summary, the primary contributions of this study are as follows:

- We propose a novel hierarchical diffusion-based (HDPose) method that can converged to a fast and accurate 3D pose by aggregating spatio-temporal information to all layers of the denoising model.
- We performed experiments with various conditioning methods, including simple 2D pose, non-hierarchical, pre-hierarchical, and post-hierarchical structure. Through empirical observation, we identified the model structure that yielded the best performance.
- Our proposed hierarchical model, when compared to state-of-the-art methods, demonstrated competitive results. It maintained a lightweight model, showcasing its effectiveness on the Human3.6M and MPI-INF-3DHP datasets.

## 2. Related Work

### 2.1. 3D Human Pose Estimation

Monocular 3D human pose estimation methods can typically be divided into two categories: frame-based approaches and sequence-based approaches. Many prior approaches to frame-based methods have employed either a CNN to directly regress the 3D pose from the image [17,18,19] or an off-the-shelf 2D pose detector to predict the 2D pose, followed by a lifting process to obtain the 3D pose, e.g., [9,10,20,21,22,23,24]. Pavlakos et al. [25] directly regressed the 3D coordinates of each joint in a pose by transforming the pose into a 3D volume and training a CNN to predict the likelihood of each joint being located within each voxel of the volume. Martinez et al. [10] proposed a method to detect 2D poses and then lifted them to 3D poses to ensure that they were less sensitive to changes in background and lighting.

The sequence-based method predicts a consistent 3D pose by using temporal information from a sequence of 2D human poses, e.g., [7,8,26,27,28,29]. Pavllo et al. [11] proposed a fully convolutional neural network architecture that utilized dilated temporal convolutions over 2D keypoints to estimate the 3D pose in a video sequence. Zheng et al. [7] proposed a transformer-based approach for accurate 3D human pose estimation from videos.

In contrast to the frame-based approach, the sequence-based approach utilizes temporal information to infer consistent 3D poses even when body joints are occluded in individual frames. The current state-of-the-art model by Zhang et al. [16] further enhances performance through a sequence-to-sequence (seq2seq) approach built upon the Transformer architecture. This advancement addresses limitations found in the previously proposed seq2frame approach. The seq2frame method, which predicts individual frames based on long-range temporal information processed by the transformer, necessitates the repetitive input of 2D keypoint sequences with substantial overlap to deduce the 3D pose for all frames. This leads to the issue of redundant computations. To resolve this challenge, Zhang et al. [16] introduced a novel transformer-based 3D human pose estimation method utilizing the seq2seq approach, enabling the prediction of consecutive frames more efficiently. We were inspired by the work of Zhang et al. [16]. Our approach integrates transformer-based 3D human pose estimation to effectively harness long-range temporal information within videos. We adopt a seq2seq approach, building upon the foundations laid by Zhang et al. [16]. This allows us to more accurately capture the nuances of human motion over extended periods, enhancing the overall effectiveness of our pose estimation framework.

### 2.2. Generative 3D Human Pose Estimation

Generative methods have emerged as a promising approach for addressing the challenges of occlusion and depth ambiguity in 3D human pose estimation. Recently, methods using many generative models, such as GAN [30], CVAE [31], and Normalizing Flows [32], have been proposed. Barsoum et al. [33] proposed a novel sequence-to-sequence model for probabilistic human motion prediction. Sharma et al. [34] addressed the ambiguity of 2D-3D lifting by generating multiple 3D posture possibilities. Wehrbein et al. [35] explored ambiguous 2D-3D inverse problems using a regularized flow-based approach with deterministic 3D-2D mapping and uncertainty modeling from 2D detectors.

Recently, methods utilizing diffusion have been proposed. Diffpose [36] considered the cross-correlation between the joints, which was not considered as a condition in previous studies. Using an embedding transformer as a condition, it is provided as a condition to the diffusion model through a joint-wise embedding vector. The study by Choi et al. [13] uses GCN [37] as a denoising model that captures the spatial anatomy of the person well, and utilizes 2D keypoints as a condition. However, this work is only optimized for frame-by-frame 3D HPE operations, which has the limitation of not exploiting important temporal trajectories in video sequences and thus does not achieve high performance. To improve on this, the work of Rommel et al. [14] utilizes spatial and temporal context by using pre-trained models as conditionals, but this method also requires significant time for convergence to the desired pose. Therefore, we provide a hierarchical aggregation of spatial and temporal context using pre-trained conditionals to enable faster convergence.

## 3. Proposed Method: Hierarchical Diffusion 3D Human Pose Estimation (HDPose)

### 3.1. Diffusion Model

The diffusion model learns to gradually denoise a sampled 3D pose starting from pure noise. A diffusion process can be divided into two processes: forward and reverse processes.

- **Forward Process** can be modeled as a Markov chain [38] wherein Gaussian noise is gradually added to the ground truth 3D pose $x_{0}$ at each subsequent step *t* until the state attains a Gaussian distribution. It is denoted as N(0,I). To train the diffusion model to denoise a 3D pose in a progressive manner, it must be provided with supervisory signals in the form of ground truth distributions. We can generate samples from these distributions using the forward diffusion process iteration, starting with the ground truth 3D pose distribution and gradually adding noise. This process can predefine $q(x_{1:T}|x_{0})$ through variance noise scheduler $\beta_{t}$ and step *t* as follows:
  (1) $$q\left(x_{1:T}|x_{0}\right)=\prod_{t=1}^{T}q\left(x_{t}|x_{t-1}\right)$$
  (2) $$q\left(x_{t}|x_{t-1}\right):=N\left(x_{t};\sqrt{1-\beta_{t}}x_{t-1},\beta_{t}I\right)$$
  We used the cosine noise variance schedule [39] to control the amount of noise added to the 3D pose at each step of the diffusion process. We enabled a reparameterization trick to make the diffusion process more efficient by enabling direct sampling from the noise distribution. Following DDPM [12], this process can be expressed as:
  (3) $$x_{t}:=\sqrt{{\bar{\alpha }}_{t}}x_{0}+\sqrt{1-{\bar{\alpha }}_{t}}\epsilon$$
  where $\alpha_{t}:=1-\beta_{t}$, ${\bar{\alpha }}_{t}:=\prod_{s=1}^{t}\alpha_{s}$ and ϵ∼N(0,I) Gaussian noise ϵ. We can optimize L by randomly sampling *t* during training, thereby exploiting these properties.
- **Reverse Process** is a process of reconstruction of the correct 3D pose from an incorrect 3D pose. The task of accurately reconstructing a 3D pose from a random distribution remains a significant challenge. To address this, we adapted the diffusion process based on the context information derived from the 2D sequence. This approach ensured the attainment of a deterministic 3D pose that aligns with the spatial and temporal embedding vectors. Reverse processes can also be expressed as a joint distribution $p_{\theta }\left(x_{t-1}|x_{t}\right)$, which describes the probability of observing a 3D pose $x_{t-1}$ at timestep *t*.
  (4) $$p_{\theta }\left(x_{t-1}|x_{t}\right):=N\left(x_{t-1};\mu_{\theta }\left(x_{t},c,t\right),\Sigma_{\theta }\left(x_{t},c,t\right)\right)$$
  In the DDPM [12], $\Sigma_{\theta }\left(x_{t},c,t\right)$ was fixed as constant. Considering the mean parameter $\mu_{\theta }\left(x_{t},c,t\right)$, we can compute the distribution of the previous timestep $x_{t-1}$ using the $\mu_{\theta }$ function, which is defined as follows.
  (5) $$\mu_{\theta }\left(x_{t},c,t\right):=\frac{1}{\sqrt{\alpha_{t}}}\left(x_{t}-\frac{\beta_{t}}{\sqrt{1-{\bar{\alpha }}_{t}}}\epsilon_{\theta }\left(x_{t},c,t\right)\right)$$
  Therefore, the only remaining task is to predict $\epsilon_{\theta }$. However, in the above method, timestep *t* is typically set to a value greater than 100 to ensure that the model can accurately learn the diffusion process. As a result, this can make the reverse diffusion process computationally expensive. Instead of predicting the noise, we approximated the reverse diffusion process using DDIM [40] to reduce the computational cost, which required fewer iterations. Therefore, we directly predicted the correct 3D pose ${\tilde{x}}_{0}$ from the trained network.
  (6) $${\tilde{x}}_{0}=f_{\theta }\left(x_{t},c,t\right)$$

### 3.2. Training and Sampling Process

**Training process.** Initially, we randomly selected a timestep t∼U(1,…,T) and sample noise ϵ∼N(0,I). $x_{t}$ was obtained by gradually adding noise to the ground truth 3D pose, with the noise level being dependent on the specific timestep *t*. We processed the pose denoising by inputting $x_{t}$, condition *c*, and timestep *t* into the denoising model $f_{\theta }$, as defined in Equation (6). This model was responsible for reconstructing the predicted 3D pose ${\tilde{x}}_{0}$. Subsequently, we applied gradient descent steps to Equation (7) until convergence to the correct 3D pose was achieved:

(7) $$L=E_{t∼\left[1,T\right],x_{0},c}[{\left\|x_{0}-{\tilde{x}}_{0}\right\|}^{2}]$$

Throughout the training, the entire diffusion process is supervised. We optimize the denoising model using a mean squared error (MSE) between the ground truth 3D pose and the predicted 3D pose.

**Sampling process** involves estimating the correct 3D pose using the trained denoising model $f_{\theta }$. We initiated the process by sampling the initial 3D pose $x_{0,T}$ from a Gaussian noise distribution corresponding to timestep *T*. The pose $x_{0,0}$ was directly predicted from $x_{0,t}$ and then fed into the denoising model to produce the incorrect 3D pose $x_{0,t-1}$ for the subsequent timestep. This procedure is described by the following equation, which outlines the DDIM [40] process:

(8) $$x_{0,t-1}=\sqrt{{\bar{\alpha }}_{t-1}}\cdot {\tilde{x}}_{0,0}+\sqrt{1-{\bar{\alpha }}_{t-1}-\sigma_{t}^{2}}\cdot \epsilon_{t}+\sigma_{t}\epsilon$$

(9) $$\epsilon_{t}=\frac{x_{0,t}-\sqrt{{\bar{\alpha }}_{t}}\cdot {\tilde{x}}_{0,0}}{\sqrt{1-{\bar{\alpha }}_{t}}}$$

In accordance with Equation (3), $\epsilon_{t}$ is derived. As $\sigma_{t}$ nears zero, its determinate nature intensifies. We commence at timestep T with $x_{0,T}$ and recursively predict the 3D pose for the next timestep using the denoising model defined in Equation (6). At each timestep, the predicted 3D pose serves as the input for the denoising model, facilitating the prediction of the subsequent 3D pose. This process is iteratively conducted *N* times, where *N* belongs to the range [1, *T*] and satisfies N<T.

### 3.3. Pre-Trained Model of Conditioning

In general, obtaining a correct 3D pose solely from the incorrect 3D data derived from a Gaussian distribution is challenging. To address this, we integrated additional conditions to more precisely steer the 3D pose reconstruction process. Nevertheless, the denoising process, when implemented using only basic 2D pose conditions, cannot achieve optimal results. The reasons for the suboptimal results when using only basic 2D pose conditions in the denoising process are as follows. First, utilizing simple 2D poses fails to effectively capture temporal information, which leads to an inability to accurately predict the appropriate trajectories for each joint, thus not achieving the best possible performance. Second, even when context information containing spatial–temporal data is provided to enhance the process, there is an issue with the prolonged time required for convergence to the optimal pose. When using a transformer as the backbone of a condition model, the self-attention mechanism has the ability to effectively capture long sequences. While this is an advantageous property for modeling complex sequences, it does require significant computational resources. This computational intensity comes from computing the interactions between all the elements in the sequence, and for the 3D HPE task, using long-range associations of the input sequence to capture temporal information between sequences improves accuracy, but requires computational complexity and significant memory to store the attention weights.

Therefore, we use a pre-trained [41] spatio-temporal transformer as the condition model. This method helps save learning time and resources and plays an important guiding role for more accurate 3D pose estimation. We pass the spatial and temporal context to the diffusion model in a hierarchical manner, which allows the diffusion model to converge faster.

### 3.4. Hierarchical Conditioning Diffusion for 3D Human Pose Estimation

**Figure 1:**
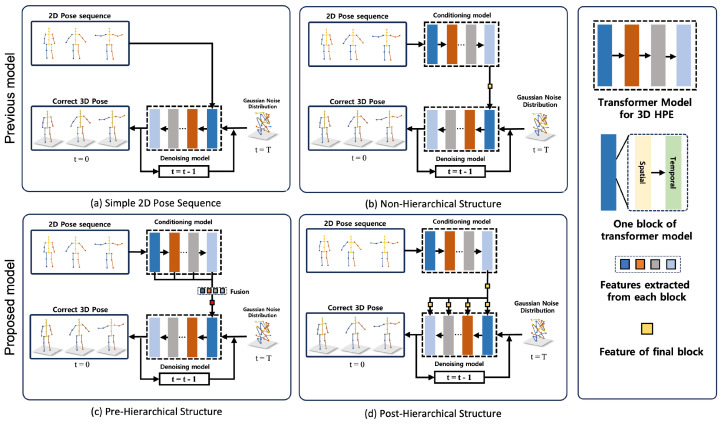
An overview of various conditioning structures. This framework comprises 4 distinct conditioning structures. (**a**) In the Simple 2D Pose Structure, prior to the diffusion process, $x^{3D}$ sampled from the Gaussian distribution and simple $x^{2D}$ are concatenated and used as the input. (**b**) The Non-Hierarchical Structure aims to improve upon the limited performance of simple $x^{2D}$ by extracting spatial-temporal context information. This information is then concatenated with $x^{3D}$ for use as input. (**c**) The Pre-Hierarchical structure aggregates $x^{3D}$ and the output $E_{F}^{2D}$ from each layer, incorporating both low-level and high-level information via a fusion module. (**d**) The Post-Hierarchical structure is a denoising model that utilizes the holistic representation $E^{2D}$. In this structure, $x^{3D}$ is projected to a higher dimension via linear projection. Next, It feeds spatial and temporal encoders with hierarchical conditioning aggregation. This process is repeated N times, ultimately converging to a refined 3D pose when t = 0.

This section presents an overview of conditional diffusion applied to 3D HPE, as illustrated in Figure 1.

**Figure 2:**
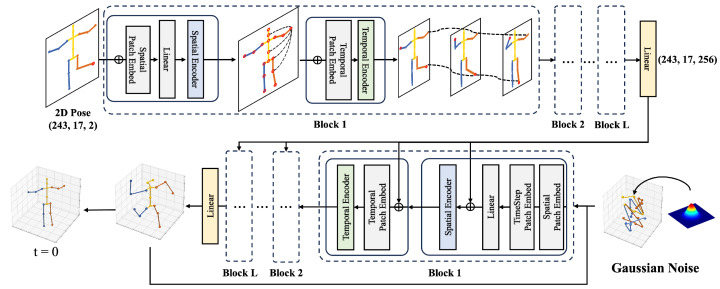
Detailed architecture of the post-hierarchical structure method. Beginning with the condition model, the 2D pose with dimensions (243, 17, 2) undergoes a linear projection, transforming its dimensions to (243, 17, 512). After being processed multiple times through the spatial and temporal encoders, a dimensional transformation to (243, 17, 256) is carried out. This allows the resulting features to be integrated with each corresponding spatial and temporal encoder within the denoising model.

- **Pre-Hierarchical Structure**
  In Figure 1c, this architecture is inspired by the work of Sun et al. [42]. Their work showed that connecting feature maps of varying depths allows networks to integrate and utilize multiscale information, which can lead to a more nuanced understanding of the input data. Instead of using a simple 2D pose as a condition, we use an $E^{2D}$ consisting of a spatial encoder $E^{S}$ and a temporal encoder $E^{T}$. The spatial encoder $E^{S}$ learns the spatial correlations between all joints in the $i_{th}$ frame. This approach allows the model to gain a more accurate understanding of the actual structure of the body and the natural connections between joints, leading to more precise and realistic pose estimation. Initially, the 2D pose $x^{2D}$ is transformed into a higher-dimensional embedding vector $X\in R^{F\times J\times D}$ via linear projection. This vector *X* is combined with the learnable spatial location sign $E_{s}\in R^{F\times J\times D}$ and then input to $E^{S}$. The output of $E^{S}$ is processed by $E^{T}$, which captures the joint correlation of each frame. As observed in [16], there are significant differences in the motion trajectories of the joints from frame to frame, so it is essential to learn a distinct trajectory pattern for each joint in each frame.
  $$E_{F}^{2D}=Concat\left(E_{1}^{S},E_{1}^{T},\ldots E_{L}^{S},E_{L}^{T}\right)W_{F}$$
  The depth of the encoder is denoted by *L*. In this framework, the fusion module uses a linear projection to align the dimensions of the concatenated features $\left(E_{i}^{S},E_{i}^{T}\right)$ with the dimensions of the spatial encoder used for denoising. In the denoising model $f_{\theta }\left(E_{F}^{2D},x_{t}^{3D},t\right)$, the input features include both the condition $E_{F}^{2D}$ and the incorrect 3D pose $x_{t}^{3D}$ and the time interval *t*. The incorrect 3D pose is then merged with the associated condition feature $E_{F}^{2D}$ and jointly trained. The training process described in Section 3.2 is then performed.
- **Post-Hierarchical Structure**
  As shown in Figure 1d, we introduce a post-hierarchical structure as an efficient way to accurately and quickly guide the construction of the correct 3D pose. From our observations, we found that spreading the final extracted features in a hierarchical structure to each encoder layer of the denoising model yields the most effective results. Similar to (b), we extract $E^{2D}$ from the final layer of the condition model. We then pass this feature, which covers the entire spatial and temporal information, to the denoising model $f_{\theta }\left(E^{2D},x_{t}^{3D},t\right)$. In traditional 3D HPE methods using diffusion, it is common to associate the condition with $x_{3D}$ only in the initial encoder, whereas our approach incorporates it in all encoder layers. The incorrect 3D pose $x_{t}^{3D}$ is transformed into a high-dimensional embedding vector, which is then added along with a spatial position embedding $E_{s}$ and a time interval embedding *t*. At each encoder step, this embedding vector is further aggregated with the global features of the condition model $E^{2D}$ to produce global condition information. The output of the spatial encoder $E^{S}$ is combined with the temporal position embedding $E_{t}$. The combined features are then reshaped in the $R^{J\times F\times D}$ dimension and provided as input to the temporal encoder $E^{T}$. This procedure is repeated on all encoder layers to the final depth to extract the final 3D pose $x_{0:t}$. The $x_{t}^{3D}$ is utilized to generate a noisy 3D pose $x_{0:t-1}$ to be input to the denoising model as the next step, which is input via DDIM [40]. This procedure is repeated *N* times. The goal is to progressively refine the pose to an accurate 3D structure. This process is repeated *N* times, progressively refining the pose to an accurate 3D reconstruction. Figure 2 shows the detailed architecture of the Post-Hierarchical Structure.

## 4. Experiment Results

### 4.1. Datasets and Evaluation Metrics

We conducted our experiments using datasets commonly adopted in previous studies. To perform a comprehensive evaluation of our proposed method, we used well-known benchmark datasets for human pose estimation: Human3.6M [43,44] and MPI-INF-3DHP [45].

**Human3.6M** is a comprehensive and challenging dataset for 3D human pose estimation in indoor environments. The dataset was captured using four synchronized high-resolution cameras operating at 50 Hz, providing high-quality data for a wide range of human poses and activities. Our model is trained on five subjects (S1, S5, S6, S7, S8) and evaluated on two subjects (S9, S11).

**MPI-INF-3DHP** is a widely used large-scale dataset for 3D human pose estimation, involving both indoor and complex outdoor scenarios. It features eight actors performing eight distinct activities captured from 14 synchronized cameras. The dataset comprises over 1.3 million frames meticulously collected from these cameras. We split a training set containing eight subjects and a test set containing seven subjects, the same as in the previous study [7,8,16].

**Evaluation Metrics.** The performance of the proposed method was evaluated using the same metrics as in previous methods. First, the proposed models were evaluated on Human3.6M using standard protocols (i.e., Protocol 1, Protocol 2). Protocol 1 used mean per-joint position error (MPJPE), which is the average of the Euclidean distance in millimeters between the ground truth pose and the predicted pose. Protocol 2 used P-MPJPE, applied to the alignment between the ground truth pose and the predicted pose. The MPI-INF-3DHP reports the percentage of correct keypoints (PCK), Area Under the Curve (AUC), and MPJPE as evaluation metrics.

### 4.2. Implementation Details

All experiments were conducted on individual NVIDIA GeForce RTX 3090 GPUs (Geforce RTX 3090 GPUs is developed by NVIDIA, based in Santa Clara, CA, USA). We employed a batch size of 4 and performed 300 epochs in training. The initial learning rate was set to 0.001 and weight decay was 7 × 10^−6^ per epoch. The Adam optimizer was used for optimization. Data augmentation was performed by applying flipping and horizontal transformations. The experiments used the PyTorch framework and the method proposed by Zhang et al. [16] as the backbone for both the condition and denoising models. The spatial and temporal encoders each had a depth of 8 and multi-head attention of 8. The embedding dimension was set to 256. As a hyperparameter for diffusion, the number of hypotheses *H* was set to 1, β started at 0.99 and decreased to 0.01, and cosine noise variance schedule [39] was used. We experimented with a timestep of *T* 1000.

### 4.3. Quantitative Results

**Figure 3:**
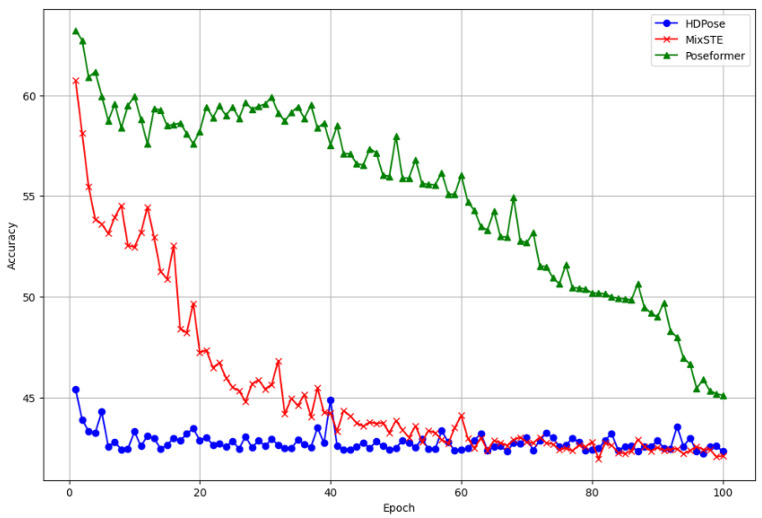
Comparison of convergence speeds across state-of-the-art models.

**Figure 4:**
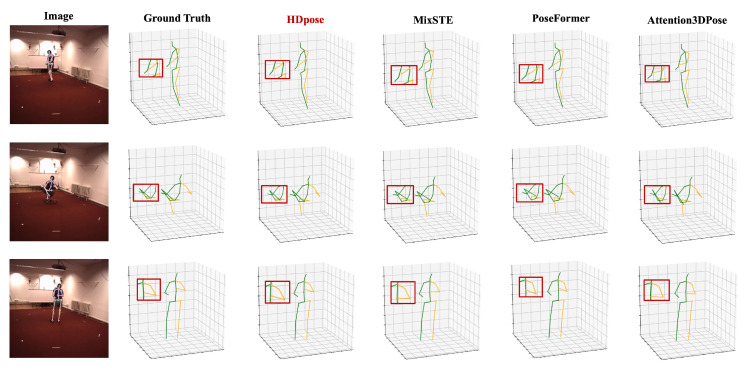
Qualitative comparison of the proposed HDPose method with other SOTA approaches using the Human3.6M.

- **Results on benchmark dataset.** Table 1 demonstrates that our method yielded nearly identical results to the previous SOTA models in terms of average MPJPE for Protocol 1 at 41.0 mm and for Protocol 2 at 32.8 mm, while outperforming other models. Specifically, on the Human3.6M, our model exhibited an improvement of approximately 4.21% (1.8 mm) for Protocol 1 and 4.65% (1.6 mm) for Protocol 2, compared to the method proposed by Shan et al. [46]. On the MPI-INF-3DHP, it exhibited a significant enhancement of 38.45% (from 58.0 mm to 35.7 mm) compared to the method proposed by Liu et al. [8]. For detailed information, refer to Table 2.
- **Results on computational complexity.** To evaluate the computational complexity of our model, we compared the number of trainable parameters with those of previous models. Despite its lightweight design, our model matched the performance of SOTA models, with a reasonable number of floating-point operations per second (FLOPs). The training process performed on a single GeForce 3090 GPU completes 100 epochs in about 24 h. Further emphasizing its efficiency, a comparison of frames per second (FPS) during the inference process revealed real-time capabilities. These detailed results are elucidated in Table 3.
- **Results on comparison of convergence.** We compared the convergence speed of our proposed method HDPose and the state-of-the-art model [7,16], and the result is reported in Figure 3. Comparing the optimal MPJPE performance over 100 epochs, Zheng et al. [7] achieved 45.1 mm in 100 epochs and Zhang et al. [16] achieved 42.2 mm in 96 epochs. On the other hand, we can see that our proposed model converges to 42.1 mm already in 60 epochs with a faster learning process. By comparing the convergence speed with other state-of-the-art (SOTA) models, we found that our proposed model outperforms others by up to 26% at peak performance. We found that our model has a faster learning convergence speed than other models using pre-trained conditioning models. The model was trained to recognize weights that were optimized to recognize features that were already useful in a spatial and temporal context.
- **Results on visualization.** Figure 4 presents a comparison of the state-of-the-art (SOTA) methods [7,16,28] and HDPose by visualizing their performance across three actions: Sitting, Greeting, and WalkingDog in Subject S11 of Human3.6M. We found that our proposed method generated more plausible poses than previous works and closely resembled the ground truth 3D pose. We also presented visualization results on MPI-INF-3DHP and on 3DPW [47], an “in-the-wild” dataset that reflects a real-world environment with varying lighting, backgrounds, and camera angles. More details on this can be found in the Appendix A.

### 4.4. Ablation Study

Using pytorch profiler [54], we found that the total time taken to perform a CUDA operation or task was 0.028 ms. We also analyzed the computational complexity according to the embedding dimension, as shown in Table 4. We fixed the embedding dimension of the conditioning model ($d_{c}$) to 256 as the embedding dimension of the pre-trained model, and compared the number of parameters and FLOPs with MPJPE while changing the embedding dimension of the denoising model ($d_{d}$), and found the most optimized performance at 256. Comparing the FPS and CUDA computation speed of our model with the 5.0 M of memory that can run on resource-intensive embedded devices, we believe that our model is suitable for use on embedded devices such as robots. This evidence strongly indicated that the proposed method was lightweight and efficient and rapidly converged to accurate 3D poses, thereby offering competitive performance in the realm of 3D HPE.

**Figure 5:**
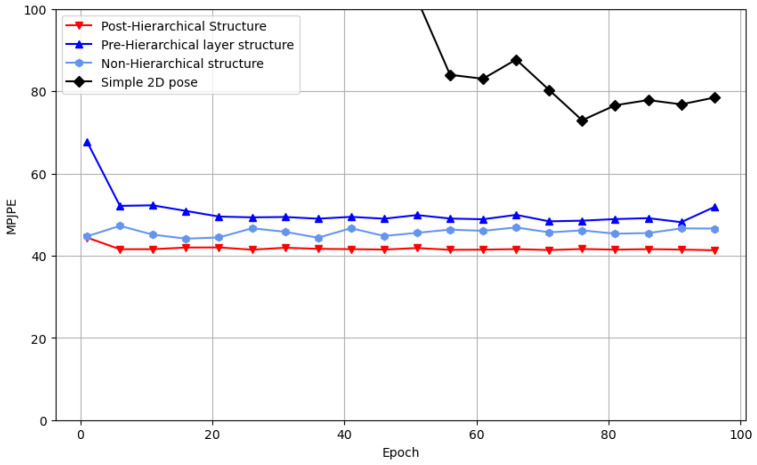
Ablation experiments based on different conditioning methods.

**Performance differences in condition.** Conditioning to demonstrate that the post-hierarchical structure achieves the highest performance and efficiency, we compared four different condition models for 100 epochs on the Human3.6M dataset with the same hyperparameter settings, including the encoder depth of 8, and one hypothesis. The post-hierarchical structure exhibited a significant improvement in MPJPE compared to the simple 2D pose (43.29%, 72.3 mm → 41.0 mm), pre-hierarchical structure (9.69%, 45.4 mm → 41.0 mm), and non-hierarchical structure (3.53%, 42.5 mm → 41.0 mm). The detailed results are presented in Figure 5.

**Figure 6:**
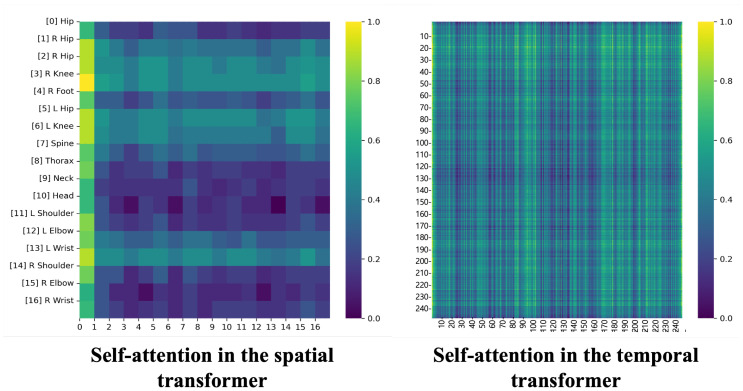
Visualization of spatial and temporal self-attention matrix. Each row and column shows the index of the joint and frames. The left side indicates the number of joints. On the right side, the figure denotes the number of frames.

**Visualization of self-attention matrix among joints and frames.** We visualize the spatial and temporal attention of the denoising model. As shown in Figure 6, the left side is the attention matrix to understand the correlation between each joint, demonstrating the capability of the model to distinguish between the left and right sides of a joint, and the right side is the attention matrix between frames, normalized to a value in the range of [0, 1]. It is easy to observe that our model has learned the connectivity between joints, even when they are physically far apart, and is also good at learning long-range associations of the input sequence.

**Figure 7:**
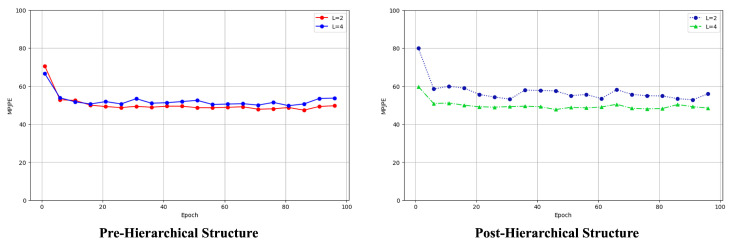
Ablation study of conditioning depth on a hierarchical structure.

**Analysis depending on hierarchical structure.** We compared the effect of hierarchical methods on accuracy to assess the performance of the proposed model at various depths of conditioning models. We observed a decline in the performance of the pre-hierarchical structure with increasing depth. Conversely, the performance of the post-hierarchical structure improved at deeper levels. These findings are detailed in Figure 7. Considering that the model’s input was a 2D pose, the integration of low-level and high-level conditioning models did not significantly enhance the 3D pose reconstruction. Consequently, we opted for a hierarchical broadcasting approach for denoising, which was centered on the final comprehensive representation of the 2D pose. This method was found to surpass existing techniques in terms of effectiveness.

**Spatio-temporal encoder of each component.** In our approach to hierarchically integrate the final comprehensive representation into a denoising model, we analyzed the performance variances when aggregating solely the spatial encoder, the temporal encoder, or a combination of both. These results are detailed in Table 5. It was observed that aggregating all spatial encoders reduced the performance compared to the use of concatenation methods. Aggregation of only the temporal encoder exhibited performance on par with employing only the spatial encoder. However, the highest performance was achieved when both the spatial and temporal encoders were aggregated together. Ultimately, the model attained its peak performance through the aggregation of all spatial and temporal encoders.

### 4.5. Limitations and Discussion

**Figure 8:**
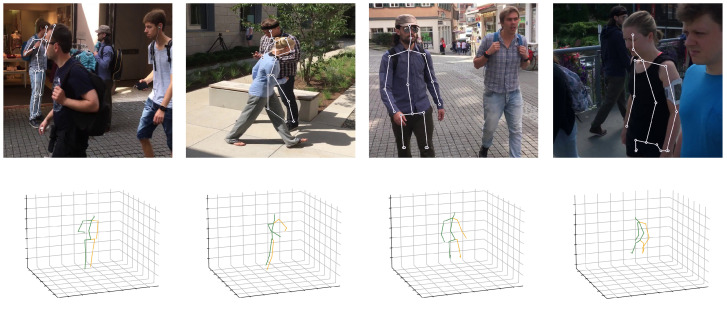
Failure cases caused by multi-person overlapping and image cropping.

Our method still has some unsolved problems, and an example of some failure cases is shown in Figure 8. We perform 3D human pose estimation for a single person. However, when many people pass by, they may overlap and be recognized as one person, making the keypoints indistinguishable from each other. We designed a model that contains spatial and temporal information as a condition model, which is passed to the diffusion model to make the model more robust to occlusion. Here, the condition acts as a guide for the diffusion model to restore the correct pose. Conversely, if it estimates the wrong pose, it will reconstruct the wrong pose. Due to the nature of the camera, the image size is limited, so if a part of the person is cropped out of the image, the temporal information is not available and the model cannot handle severe occlusion.

## 5. Conclusions

In this paper, we proposed HDpose, a new framework designed for hierarchical conditioning in diffusion-based 3D human pose estimation. When performing 3D human pose estimation with a diffusion model, the simple use of a 2D pose as the condition necessitates several steps to converge to the correct 3D pose, often resulting in suboptimal performance. Therefore, we emphasized the need for a method that rapidly converged to the correct 3D pose. HDpose leveraged a condition model to generate a holistic representation, which was then aggregated across all layers of the denoising model to ultimately converge to the correct 3D pose. By comparing the convergence speed with other state-of-the-art (SOTA) models, we found that our proposed model outperforms others by up to 26% at peak performance. When evaluated against two widely used benchmark datasets in comparison with state-of-the-art (SOTA) methods, our model demonstrated equivalent performance while significantly reducing the model size by approximately 85.12%. Thus, a more lightweight model was realized.

## Figures and Tables

**Table 1:** Quantitative evaluation of 3D human pose estimation methods using the standard evaluation metrics MPJPE (mm) and PA-MPJPE(mm) on the Human3.6M dataset. Part of the data in Table 1 was referenced from the respective papers [13,16,36,48]. (‡)—using the diffusion method. Bold: best.

Protocol 1 (MPJPE)	Dir.	Disc	Eat	Greet	Phone	Photo	Pose	Purch.	Sit	SitD.	Smoke	Wait	WalkD.	Walk	WalkT.	Avg.
Zhao et al. [49]	45.2	50.8	48.0	50.0	54.9	65.0	48.2	47.1	60.2	70.0	51.6	48.7	54.1	39.7	43.1	51.8
Cai et al. [50] (*N* = 7)	44.6	47.4	45.6	48.8	50.8	59.0	47.2	43.9	57.9	61.9	49.7	46.6	51.3	37.1	39.4	48.8
Pavllo et al. [11] (*N* = 243)	45.2	46.7	43.3	45.6	48.1	55.1	44.6	44.3	57.3	65.8	47.1	44.0	49.0	32.8	33.9	46.8
Liu et al. [28] (*N* = 243)	41.3	43.9	44.0	42.2	48.0	57.1	42.2	43.2	57.3	61.3	47.0	43.5	47.0	32.6	31.8	45.1
Zeng [27]	46.6	47.1	43.9	41.6	45.8	49.6	46.5	40.0	53.4	61.1	46.1	42.6	43.1	31.5	32.6	44.8
Shan et al. [51] (*N* = 243)	40.8	44.5	41.4	42.7	46.3	55.6	41.8	41.9	53.7	60.8	45.0	41.5	44.8	30.8	31.9	44.3
Zheng et al. [7] (*N* = 81)	41.5	44.8	39.8	42.5	46.5	51.6	42.1	42.0	53.3	60.7	45.5	43.3	46.1	31.8	32.2	44.3
Chen et al. [52] (*N* = 243)	41.4	43.2	40.1	42.9	46.6	51.9	41.7	42.3	53.9	60.2	45.4	41.7	46.0	31.5	32.7	44.1
Li et al. [8] (*N* = 351)	39.2	43.1	40.1	40.9	44.9	51.2	40.6	41.3	53.5	60.3	43.7	41.1	43.8	29.8	30.6	43.0
Shan et al. [46] (*N* = 243)	38.9	42.7	40.4	41.1	45.6	49.7	40.9	39.9	55.5	59.4	44.9	42.2	42.7	29.4	29.4	42.8
Zhang et al. [16] (*N* = 243)	37.9	**40.7**	37.8	**39.6**	**42.3**	**50.2**	39.9	39.9	**51.6**	**55.6**	42.1	39.9	**40.8**	**27.9**	**28.0**	**40.9**
Choi et al. [13] (*H* = 10) ‡	43.4	50.7	45.4	50.2	49.6	53.4	48.6	45.0	56.9	70.7	47.8	48.2	51.3	43.1	43.4	49.4
Holmquist et al. [36] (*H* = 200) ‡	38.1	43.1	**35.3**	43.1	46.6	**48.2**	**39.0**	**37.6**	51.9	59.3	**41.7**	47.6	45.4	37.4	36.0	43.3
Ours (*N* = 243, *H* = 1)	**37.8**	**40.7**	37.7	**39.6**	42.4	50.2	39.8	40.2	51.8	55.8	42.2	**39.8**	41.0	**27.9**	28.1	41.0
Protocol 2 (PA-MPJPE)	Dir.	Disc	Eat	Greet	Phone	Photo	Pose	Purch.	Sit	SitD.	Smoke	Wait	WalkD.	Walk	WalkT.	Avg.
Cai et al. [50] (*N* = 7)	35.7	37.8	36.9	40.7	39.6	45.2	37.4	34.5	46.9	50.1	40.5	36.1	41.0	29.6	33.2	39.0
Liu et al. [28] (*N* = 243)	32.3	35.2	33.3	35.8	35.9	41.5	33.2	32.7	44.6	50.9	37.0	32.4	37.0	25.2	27.2	35.6
Zheng et al. [7] (*N* = 81)	32.5	34.8	32.6	34.6	35.3	39.5	32.1	32.0	42.8	48.5	34.8	32.4	35.3	24.5	26.0	34.6
Chen et al. [52] (*N* = 243)	32.6	35.1	32.8	35.4	36.3	40.4	32.4	32.3	42.7	49.0	36.8	32.4	36.0	24.9	26.5	35.0
Li et al. [8]	31.5	34.9	32.8	33.6	35.3	39.6	32.0	32.2	43.5	48.7	36.4	32.6	34.3	23.9	25.1	34.4
Shan et al. [46] (*N* = 243)	31.3	35.2	32.9	33.9	35.4	39.3	32.5	31.5	44.6	48.2	36.3	32.9	34.4	23.8	23.9	34.4
Zhang et al. [16] (*N* = 243)	30.8	33.1	30.3	31.8	**33.1**	39.1	31.1	30.5	42.5	**44.5**	**34.0**	30.8	32.7	22.1	**22.9**	32.6
Choi et al. [13] (*H* = 10) ‡	35.9	40.3	36.7	41.4	39.8	43.4	37.1	35.5	46.2	59.7	39.9	38.0	41.9	32.9	34.2	39.9
Holmquist et al. [36] (*H* = 200) ‡	**27.9**	**31.4**	**29.7**	**30.2**	34.9	**37.1**	**27.3**	**28.2**	**39.0**	46.1	34.2	32.3	33.6	26.1	27.5	**32.4**
Ours (*N* = 243, *H* = 1)	31.0	33.2	30.6	31.9	33.2	39.2	31.1	30.7	42.5	45.0	34.1	**30.7**	**32.5**	**22.0**	23.0	32.8

**Table 2:** Quantitative evaluation of 3D human pose estimation methods using the evaluation metrics PCK, AUC, MPJPE (mm) on the MPI-INF-3DHP dataset. Part of the data in Table 2 was referenced from the respective papers [7,46,48]. Bold: best.

Method		PCK↑	AUC↑	MPJPE↓
Pavllo et al. [11] (*T* = 243)	CVPR’19	85.5	51.5	84.8
Wang et al. [53] (*T* = 96)	ECCV’20	86.9	62.1	68.1
Chen et al. [52] (*T* = 25)	TCSVT’21	87.9	54.0	79.1
Liu et al. [8] (*T* = 9)	CVPR’22	93.8	63.3	58.0
Zhang et al. [16] (*T* = 243)	CVPR’22	**96.9**	**75.8**	**35.4**
Ours (*T* = 243)	Ours	96.5	75.6	35.7

**Table 3:** Analysis on computational complexity. Part of the data in Table 3 was referenced from the respective papers [16,46].

Method	MPJPE	Params (M)	FLOPs	FPS
Zheng et al. [7]	44.4	9.5	1358	269
Shan et al. [46]	42.8	6.7	1737	3040
Zhang et al. [16]	40.9	33.6	645	4547
Ours	41.0	5.0	78.5	4054

**Table 4:** Ablation study computational complexity according to embedding dimension. Embedding dimension of condition ($d_{c}$), embedding dimension of post-hierarchical model ($d_{d}$).

$d_{c}$	$d_{d}$	MPJPE	Params (M)	FLOPs
256	64	47.6	0.3	5.0
256	128	44.4	1.2	19.7
256	256	41.0	5.0	78.5
256	512	41.7	19.9	313.0

**Table 5:** Analysis of performance based on hierarchical aggregation of denoising models.

Hiearchical Aggregation	Spatial	Temporal	MPJPE
Spatial only	✓	×	45.9
Temporal only	×	✓	45.6
Spatial and temporal	✓	✓	41.0

**Figure A1:**
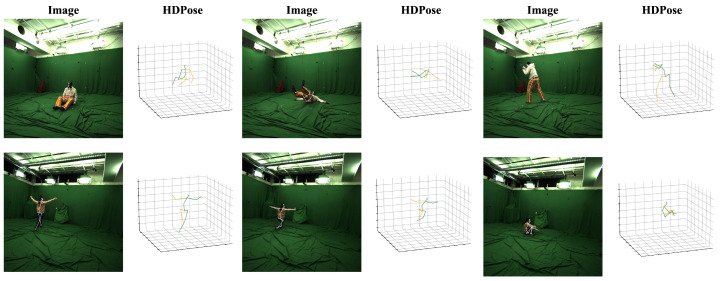
Qualitative comparison of the proposed HDPose method using the MPI-INF-3DHP.

**Figure A2:**
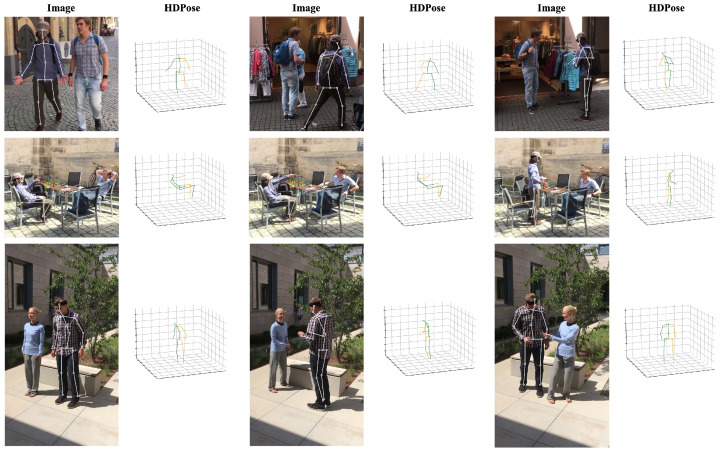
Qualitative result of the proposed HDPose method with “in the wild” using the 3DPW.

## Data Availability

The Human3.6M dataset is available upon request from the author [43]. The MPI-INF-3DHP dataset: https://vcai.mpi-inf.mpg.de/3dhp-dataset/. The 3DPW dataset: https://virtualhumans.mpi-inf.mpg.de/3DPW/.
